# Case Report and Genomic Characterization of a Novel Porcine Nodavirus in the United States

**DOI:** 10.3390/v13010073

**Published:** 2021-01-07

**Authors:** Chenghuai Yang, Leyi Wang, Kent Schwartz, Eric Burrough, Jennifer Groeltz-Thrush, Qi Chen, Ying Zheng, Huigang Shen, Ganwu Li

**Affiliations:** 1 Department of Veterinary Diagnostic and Production Animal Medicine, College of Veterinary Medicine, Iowa State University, Ames, IA 50011, USA; ychenghuai@163.com (C.Y.); kschwart@iastate.edu (K.S.); burrough@iastate.edu (E.B.); jgroeltz@iastate.edu (J.G.-T.); meadowstrain@gmail.com (Q.C.); zhy@iastate.edu (Y.Z.); hgshen@iastate.edu (H.S.); 2 China Institute of Veterinary Drug Control, Beijing 100081, China; 3 Veterinary Diagnostic Laboratory and Department of Veterinary Clinical Medicine, College of Veterinary Medicine, University of Illinois at Urbana-Champaign, Urbana, IL 61802, USA; leyiwang@illinois.edu

**Keywords:** porcine nodavirus, genome, detection, characterization, United States

## Abstract

Nodaviruses are small bisegmented RNA viruses belonging to the family *Nodaviridae*. Nodaviruses have been identified in different hosts, including insects, fishes, shrimps, prawns, dogs, and bats. A novel porcine nodavirus was first identified in the United States by applying next-generation sequencing on brain tissues of pigs with neurological signs, including uncontrollable shaking. RNA1 of the porcine nodavirus had the highest nucleotide identity (51.1%) to the Flock House virus, whereas its RNA2 shared the highest nucleotide identity (48%) with the RNA2 segment of caninovirus (Canine nodavirus). Genetic characterization classified porcine nodavirus as a new species under the genus *Alphanodavirus*. Further studies are needed to understand the pathogenicity and clinical impacts of this virus.

## 1. Introduction

Nodaviruses are small, nonenveloped isometric bisegmented RNA viruses [1]. Nodavirus was first identified in *Culex tritaeniorhynchus* mosquitoes in the Japanese village of Nodamura [2]. Subsequently, other members of the *Nodaviridae* family were identified in a variety of insects, including the Black beetle virus, Flock House virus, Boolara virus, and Pariacoto virus. In addition to insects, nodavirus was also identified as the cause of neurological disease in farmed striped jack fish in 1992, and in many other fish species. Based on the genetic diversity of the RNA2 segment, nodaviruses are taxonomically classified into two genera, *Alphanodavirus* and *Betanodavirus*, which infect insects and fish, respectively. Recently, novel nodaviruses that infect shrimps and prawns had distinct genomic sequences and thus have been tentatively proposed as the genus *Gammanodavirus* [3,4,5].

The genome of nodavirus consists of two linear, single-stranded, and positive-sense RNA molecules. The two genome segments are capped at the 5′ ends but not polyadenylated at the 3′ ends. The larger segment RNA1 is approximately 3.1 kb in length, encoding the protein A, an RNA-dependent RNA polymerase (RdRp). A subgenomic transcript RNA3 is synthesized from RNA1 and encodes two small nonstructural proteins: B1, the function of which is unknown; and B2, which functions as an RNA silencing inhibitor during replication [6,7]. Not all nodaviruses encode B1. The smaller segment RNA2 is approximately 1.4 kb long, encoding the protein α, a viral capsid protein precursor. During the viral assembly, protein α is autocleaved into a 38 kDa protein β and a 5 kDa protein γ near its C-terminus at the conserved Asn/Ala site [1].

The presence of nodavirus in mammalian hosts is not common. A bat nodavirus detected in the brain of *Eptesicus serotinus* was reported in 2014 [8], and nodaviruses have also been detected in otter and feral dog feces [9,10]. Although originally isolated from insects, nodaviruses of the genus *Alphanodavirus* were able to infect mammals in experiments. *Nodamura virus* (NoV), the prototype species of the genus *Alphanodavirus*, was able to lethally infect both insects and mammals, including suckling mice and suckling hamsters [2,11,12,13]. Therefore, NoV is the only known arthropod-borne virus that can infect insects and vertebrate animals. Serological evidence also showed that NoV might be able to infect pigs. It was reported that 13 of 16 pigs (81%) had positive neutralizing antibodies against NoV in 1956, and 14 of 27 pigs (51%) in 1957, near Tokyo, Japan [14]. However, no nodavirus has been identified and genetically characterized from naturally infected pigs. In the present study, we report identification of porcine nodavirus for the first time in the world.

## 2. Materials and Methods

### 2.1. Pig Samples

Different types of pig samples including brain, spinal cord, heart, liver, kidney, spleen, intestine, lymphoid tissues, blood, and feed were submitted by a client to the Iowa State University Veterinary Diagnostic Laboratory (ISU-VDL).

### 2.2. Testing

Real-time PCR testing was performed on *Pestivirus*, *Sapelovirus*, *Teschovirus*, *Astrovirus*, *Glaesserella parasuis*, porcine circovirus 2, porcine reproductive and respiratory syndrome virus (PRRSV), and *Streptococcus suis*. The sequences of the primers and probes used are shown in Table 1. All the real-time PCR assays were performed in a reaction mixture of 20 μL containing 5 μL TaqMan Fast Virus 1-Step Master Mix (Applied Biosystems, Waltham, MA, USA), 0.8 μL (final concentration 0.4 µM) of each of the primers, 0.4 μL (final concentration 0.2 µM) of probe, 8 μL nuclease-free water, and 5 μL extracted RNA. The amplification was performed at 50 °C for 5 min, then at 95 °C for 20 s, followed by 40 cycles at 95 °C for 3 s and 60 °C for 30 s. The PRRSV real-time RT PCR was performed with TaqMan NA and EU PRRSV Reagents (Applied Biosystems) following the manufacturer’s instructions.

### 2.3. Next-Generation Sequencing (NGS) and Sequence Analysis

Nucleic acids extracted from brain tissue were subjected to a sequence-independent, single-primer amplification, library preparation using an Illumina Nextera XT kit, and sequenced using an Illiumina MiSeq v2 sequencing kit (500 cycles) on MiSeq (Illumina, San Diego, CA, USA) as previously described [11]. Raw FastQ files were analyzed using Kraken software and assembled using a SPAdes assembler. A local BLAST (blastx) was performed on the assembler contigs. MEGA 7.0.26 was used for sequence alignment and phylogenetic tree analysis, and a BioEdit sequence alignment editor was used to calculate the sequence identity.

### 2.4. Real-Time RT-PCR for Porcine Nodavirus

The real-time RT-PCR was performed in a reaction mixture of 25 μL containing 12.5 μL 2× AgPath-ID RT-PCR Buffer (Applied Biosystems), 1 μL 25× RT-PCR Enzyme Mix, 1 μL (final concentration 0.4 µM) of each of the Noda_F and Noda_R primers (Table 1), 0.5 μL (final concentration 0.2 µM) of probe Noda_P, 4 μL nuclease-free water, and 5 μL extracted RNA. The amplification was performed at 48 °C for 10 min, then at 95 °C for 10 min, followed by 40 cycles at 95 °C for 15 s and 60 °C for 45 s. For each amplification plot, a cycle threshold (CT) value was calculated to represent the cycle number at which the reporter signal was above threshold.

## 3. Results and Discussion

In November of 2017, an acute onset of neurologic signs was observed in one barn of 20-week-old pigs on a six-barn site in North Carolina. The affected pigs had abrupt onset of full-body tremors and uncontrollable shaking, followed by prostration and death within 24 h. Affected pigs were often bullied by unaffected pigs, which likely contributed to their rapid decline and death. Twenty-two pigs died over a two-day period, and a similar disease was reported in an adjacent three barn site. Samples including brain, heart, lung, liver, kidney, spleen, tonsil, lymph node, intestine, colon, blood, and feed were submitted to ISU-VDL. Neither gross lesions nor histopathological lesions were observed in brain, spinal cord, heart, liver, kidney, spleen, intestine, and lymphoid tissues. The polymerase chain reaction (PCR) was negative for common porcine pathogens, including *Pestivirus*, *Sapelovirus*, *Teschovirus*, *Astrovirus*, *Glaesserella parasuis*, and porcine circovirus 2, using routine assays at the ISU-VDL. Although both PRRSV and *S. suis* were detected in lung tissue, findings based on lesions and clinical signs did not favor them as causes of the observed clinical signs or deaths. The differential diagnoses included various metabolic diseases and intoxications, which were then excluded.

The brain-tissue sample was subsequently analyzed using NGS technology on a MiSeq platform. Raw FastQ files was analyzed using Kraken software and assembled using a SPAdes assembler [20,21]. MEGA 7.0.26 was used for sequence alignment and phylogenetic tree analysis, and a BioEdit sequence alignment editor was used to calculate the sequence identity [22]. Kraken taxonomical analysis of raw FastQ data and local nucleotide blast of assembled contigs revealed no hit to viral reads. However, the protein BLAST (blastx) of assembled contigs revealed a low level of amino acid sequence similarity to a RdRp encoded by the RNA1 genome segment in *Nodaviridae* family. Further analysis showed that two separated segments, RNA1 (3065 nt) and RNA2 (1370 nt) (Accession numbers: MK014575 and MK014576), with similar genome structures to those of nodavirus had been assembled, and the virus was tentatively named as porcine nodavirus. The nucleotide sequence alignment revealed that the RNA1 segment of pig nodavirus showed low identities (23.4% to 51.1%) to other nodaviruses (Table 2). Surprisingly, the pig nodavirus was least closely related to the RNA1 of feral dog and bat nodaviruses (23.4% and 32.1% nt identities, respectively); instead, it had the highest nucleotide identity to the Flock House virus (51.1%). The protein A encoded by the genome segment RNA1 was 1014 amino acids in length, and contained the core motifs defined for RdRp. The protein A of porcine nodavirus shared 14.2% to 44.6% of identities with other nodaviruses at the amino acid level (Table 2).

The smaller genome segment RNA2 of the identified porcine nodavirus contained 1370 nt. Interestingly, unlike RNA1, the RNA2 of porcine nodavirus shared the highest identity (48% and 47.2%, at both nucleotide and amino acid levels, respectively) with the RNA2 segment of caninovirus (Canine nodavirus) (Table 2) [9], suggesting that porcine nodavirus could be a potential recombinant between nodaviruses of insects and mammals. The RNA2 of porcine nodavirus shared relatively higher identities (37.7–46.2%) with alphanodaviruses isolated from insects than *Betanodavirus* isolated from fishes (24.3–25.1%) and *Gammanodavirus* isolated from shrimps and prawns (19.4–29%) (Table 2). Similarly, the capsid precursor protein of porcine nodavirus encoded by RNA2 shared significantly higher similarities to those of other *Alphanodavirus* (33.2–41.5%) than *Betanodavirus* (5.9–6.9%) and *Gammanodavirus* (7–8.6%) at the amino acid level.

**Figure 1:**
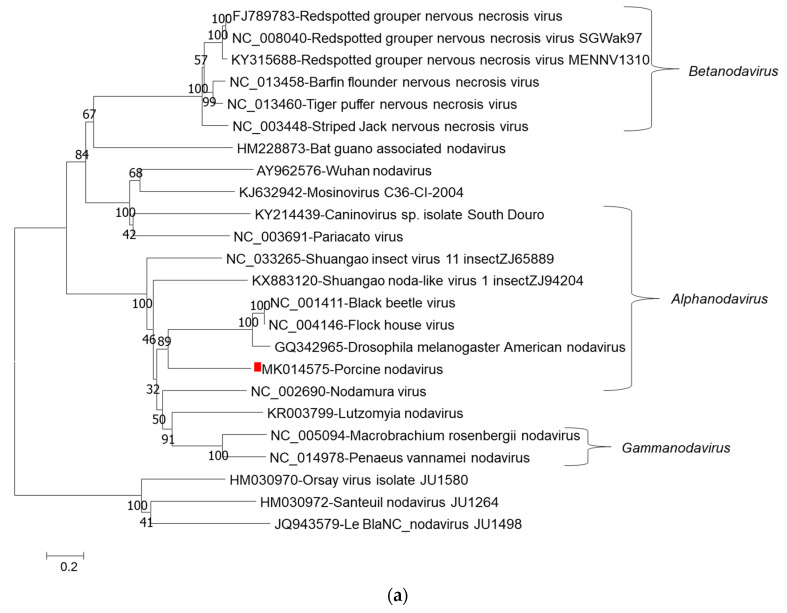

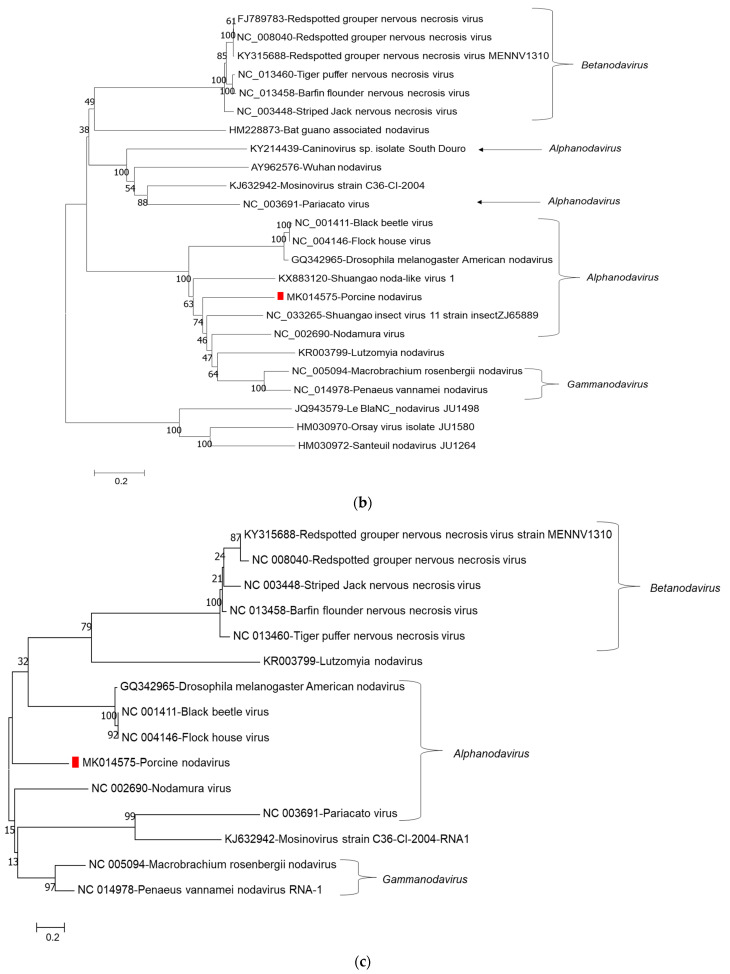
Phylogenetic relationship of porcine nodavirus (indicated by red square) with other representative nodaviruses. The tree was constructed by using the neighbor-joining method with the maximum composite likelihood model in MEGA version 7.0.26 (http://www.megasoftware.net) with 1000 bootstrap replicates, based on the nucleotide sequence of RNA1 (**a**), amino acid sequences of RNA dependent RNA polymerase (**b**), and amino acid sequences of B2 (**c**).

**Figure 2:**
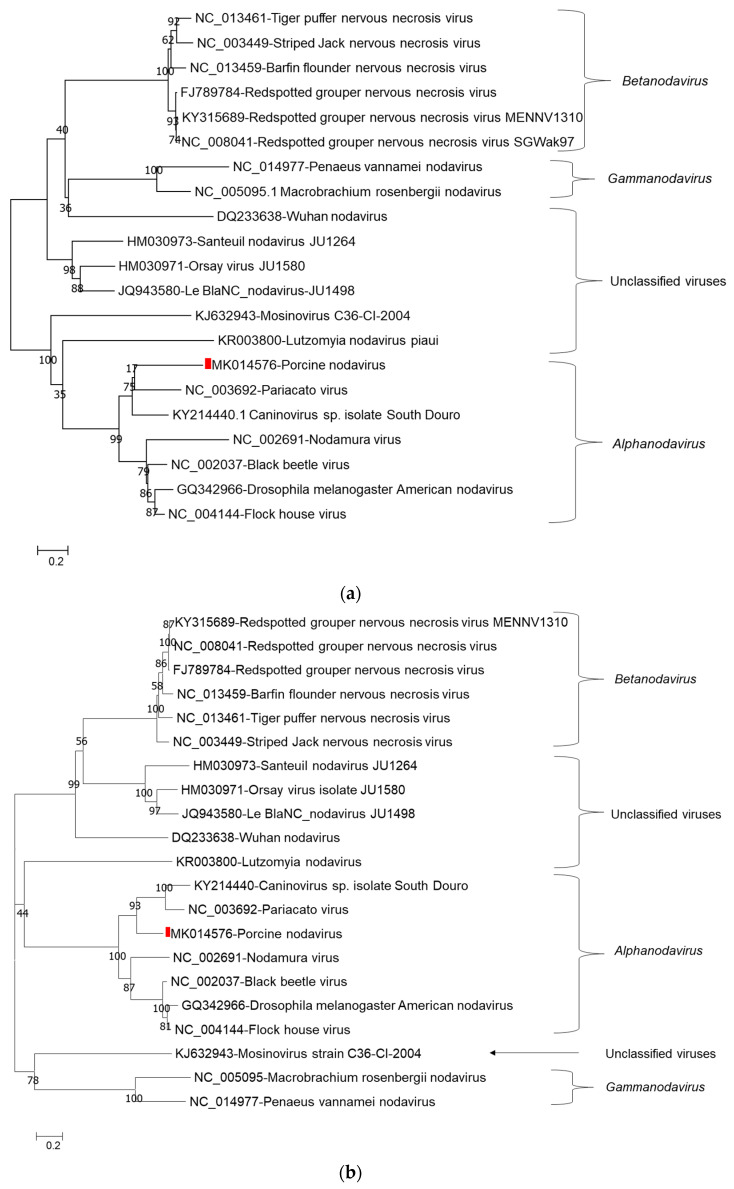
Phylogenetic relationship of porcine nodavirus (indicated by red square) with other representative nodaviruses. The tree was constructed by using the neighbor-joining method with the maximum composite likelihood model in MEGA version 7.0.26 (http://www.megasoftware.net) with 1000 bootstrap replicates, based on nucleotide sequence of RNA2 (**a**) and amino acid sequence of capsid protein precursor alpha (**b**).

Phylogenetic tree analysis of nucleotide sequences of two segments confirmed that porcine nodavirus cluster together with isolates from *Alphanodavirus*. Specifically, porcine nodavirus clustered together with three viruses (Black beetle virus, Flock House virus, and *Drosophila melanogaster* American nodavirus) and two viruses (Shuangao insect virus 11 and Nodamura virus) in the RNA 1 nucleotide and amino acid trees, respectively (Figure 1a–c); and clustered together with two viruses (caninovirus and Pariacato virus) at both the nucleotide and amino acid levels of the RNA2 segment (Figure 2a,b).

The classification of nodaviruses is based on the genetic diversity of the RNA2 segment, and a novel species can be classified if the identity of the RNA2 to the most closely related species is less than 80% and 87% at the nucleotide and amino acid levels, respectively. Porcine nodavirus shared the highest identities (<50%) of RNA2 to the feral dog isolate at nucleotide and amino acid levels (Table 2). Therefore, porcine nodavirus was classified as a novel species in the genus *Alphanodavirus*.

The fish nodavirus known as nervous necrosis virus (*Betanodavirus* genus) mainly targets the central nervous system, including the brain, spinal cord, and retina. The infected fish suffer neurological disorders characterized by viral encephalopathy and retinopathy [23,24]. In addition, the NoV, which belongs to the genus *Alphanodavirus*, could experimentally infect suckling mice leading to hind-limb paralysis [2]. The novel porcine nodavirus identified in our study was detected in the brain tissue of pigs showing neurological signs and uncontrollable shaking. The real-time RT-PCR testing detected the nodavirus in pig brain and spinal cord samples, with Ct values of 26.2 and 26.5. Future studies are required to determine the significance and potential pathogenicity of this porcine nodavirus, and epidemiological studies are needed to monitor its evolution and clinical impact.

## Figures and Tables

**Table 1:** Primers and probes used in this study.

Target Pathogen	Name	Sequence	Reference
Porcine nodavirus	Noda_F Noda_P Noda_R	TTGTCCCAACCTACAGGA FAM-CCGAATCATGCGACCACACG-BHQ TTGGCCAGTGGACTTGAA	
Porcine pestivirus	APPV_RTF APPV_RTR APPV_RTP	TGCCTGGTATTCGTGGC TCATCCCATGTTCCAGAGT FAM-CCTCCGTCTCCGCGGCTTCTTTGG-BHQ	[15]
Porcine sapelovirus	PSV_RTF PSV_RTR PSV_RTP	GGCAGTAGCGTGGCGAGC CTACTCTCCTGTAACCAGT FAM-CGATAGCCATGTTAGTG-MGB	[16]
Porcine teschovirus	PTV_RTF PTV_RTR PTV_RTP	CACCAGCGTGGAGTTCCTGTA AGCCGCGACCCTGTCA FAM-TGCAGGACTGGACTTG-MGB	[17]
Porcine astrovirus 3	PoAstV3_RTF PoAstV3_RTR PoAstV3_RTP	ATGACYCTCTATGGGAAACTCCTT GTGCCTRGCAACAACCTCCAA FAM-TTGGCCAYAACCTCCCTGA-MGB	[18]
Porcine circovirus 2	PCV2_RTF PCV2_RTR PCV2_RTP	GACTGTWGAGACTAAAGGTGGAACTGTA GCTTCTACACCTGGGACAGCA FAM-CCCGTTGGAATGGT-MGB	
*Glaesserella parasuis*	GPS_RTF GPS_RTR GPS_RTP	CCACTTACTTGAAGCCATTCTTCTT CCGCTTGCCATACCCTCTT FAM-ATCGGAAGTATTAGAATTAAGKGC-MGB	[19]
*Streptococcus suis*	Ssuis_RTF Ssuis_RTR Ssuis_RTP	CTTTTGGACAGTTTCGGAGAAGA TTTTCGTTTTCAAGAACTCGTTTG FAM-AAGACCGTTATCAGACAAC-MGB	

**Table 2:** Identity (%) of porcine nodavirus to other nodaviruses.

Strain	RNA1		RNA2		GenBank Accession #
	nt	aa	nt	aa	
Caninovirus_sp._isolate_South_Douro	23.4	14.2	48	47.2	KY214439, KY214441
Pariacato_virus	33.5	19	46.2	41.5	NC_003691, NC_003692
Flock_House_virus	51.1	40.2	39.8	32.7	NC_004146, NC_004144
*Drosophila_melanogaster*_American_nodavirus	50.3	39.9	39.7	30.3	GQ342965, GQ342966
Black_beetle_virus	51	39.8	39.2	31.8	NC_001411, NC_002037
Nodamura_virus	48	44.5	37.7	33.2	NC_002690, NC_002691
Tiger_puffer_nervous_necrosis_virus	34.5	17.5	25.1	5.9	NC_013460, NC_013461
Wuhan_nodavirus	31.3	16.3	25.1	6.5	AY962576, DQ233638
Striped_Jack_nervous_necrosis_virus	33.8	17.5	25	6.4	NC_003448, NC_003449
Redspotted_grouper_nervous_necrosis_virus	34.7	17	24.6	6.9	FJ789783, FJ789784
Redspotted_grouper_nervous_necrosis_virus_SGWak97	34.4	16.9	24.4	6.6	NC_008040, NC_008041
Barfin_flounder_nervous_necrosis_virus	34.3	17.4	24.3	5.9	NC_013458, NC_013459
Mosinovirus_C36-CI-2004	32.9	18.6	21.7	6.4	KJ632942, KJ632943
*Macrobrachium_rosenbergii*_nodavirus	46.4	42.9	29	8.6	NC_005094, NC_005095
Redspotted_grouper_nervous_necrosis_virus_MENNV1310	34.3	17.1	26	6.6	KY315688, KY315689
*Penaeus_vannamei*_nodavirus	48	42.6	19.4	7	NC_014978, NC_014977
Lutzomyia_nodavirus_piaui	45.3	34.9	18.4	4.4	KR003799, KR003800
Santeuil_nodavirus_JU1264	26.2	10.7	14.8	8.2	HM030972, HM030973
Orsay_virus_JU1580	27.4	12.3	14.2	4.2	HM030970, HM030971
Le_Blanc_nodavirus_JU1498	25.6	11.3	13.5	12.1	JQ943579, JQ943580
Bat_guano_associated_nodavirus	32.1	19.2	-	-	HM228873,
Shuangao_noda-like_virus_1_insectZJ94204	45.9	44.6	-	-	KX883120
Shuangao_insect_virus_11_insectZJ65889	45	45	-	-	NC_033265

nt: nucelotide; aa: amino acid; -: not available.

## Data Availability

The data presented in this study are available in the present article. Genome sequences were deposited in GenBank under the accession numbers MK014575–MK014576.
